# Social context facilitates visuomotor synchrony and bonding in children and adults

**DOI:** 10.1038/s41598-021-02372-2

**Published:** 2021-11-24

**Authors:** Ellen M. Howard, Danielle Ropar, Roger Newport, Bahar Tunçgenç

**Affiliations:** 1 School of Psychology, The University of Nottingham, Nottingham, UK; 2 School of Sport, Exercise and Health Sciences, Loughborough University, Loughborough, UK; 3 Institute of Cognitive and Evolutionary Anthropology, University of Oxford, Oxford, UK

**Keywords:** Psychology, Human behaviour

## Abstract

Interpersonal synchrony is a fundamental part of human social interaction, with known effects on facilitating social bonding. Moving in time with another person facilitates prosocial behaviour, however, it is unknown if the degree of synchronisation predicts the degree of social bonding. Similarly, while people readily fall in synchrony even without being instructed to do so, we do not know whether such spontaneous synchronisation elicits similar prosocial effects as instructed synchronisation. Across two studies, we investigated how context (social vs non-social stimulus) and instruction (instructed vs uninstructed) influenced synchronisation accuracy and bonding with the interaction partner in adults and children. The results revealed improved visuomotor synchrony within a social, compared to non-social, context in adults and children. Children, but not adults, synchronised more accurately when instructed to synchronise than when uninstructed. For both children and adults, synchronisation in a social context elicited stronger social bonding towards an interaction partner as compared to synchronisation in a non-social context. Finally, children’s, but not adults’, degree of synchrony with the partner was significantly associated with their feelings of social closeness. These findings illuminate the interaction of sensorimotor coupling and joint action in social contexts and how these mechanisms facilitate synchronisation ability and social bonding.

Interpersonal synchrony, whereby two or more people move in temporal and spatial coordination with each other, is observed frequently and cross-culturally in group dance, marching bands, and children’s clapping games^1,2^. Research investigating embodied cognition has shown that synchronous interpersonal movement plays a crucial role in social bonding starting from early infancy^3–6^. Interpersonal synchrony may serve important cultural-evolutionary functions by helping establish and communicate positive affect, group identity and bonding with others^7^. Distinctly from other forms of social coordination (e.g. mimicry and imitation), in interpersonal synchrony, individuals become temporally and spatially aligned in their movements rather than after a short delay. It has been shown that interpersonal synchrony is a self-organising system which can be mathematically modelled^8^. Such modelling specifies that oscillations of synchronised individuals settle either within in-phase synchrony (i.e., individuals move in the same way at the same point in the cycle) or anti-phase synchrony (i.e., individuals move with opposite movement at the same point in the cycle)—similar to the oscillations of physical metronomes. There is growing evidence to suggest that interpersonal synchrony is driven by the same coordination principles of the physical world across different contexts, underpinned by the same self-organising physical principles^9–11^. However, relatively little is known about how social versus non-social contexts impact synchronisation ability and its subsequent social bonding outcomes in children and adults. Building upon bottom–up sensory and top–down joint action accounts of interpersonal synchrony, we examined the conditions that facilitate synchronisation and its social bonding outcomes in adults (*Study 1*) and in children (*Study 2*).

Individuals can synchronise their movement with a non-social object (i.e., a metronome) or with another person (interpersonal synchrony). When moving together with another person, different degrees of shared intentionality may exist among the interacting partners. For instance, two individuals may be instructed to synchronise with each other (‘instructed synchrony’), they may be instructed to synchronise with an external metronome beat and may thus end up moving synchronously with each other ('incidental synchrony’) or they may spontaneously synchronise their movements in the absence of any instruction on how to move (‘uninstructed synchrony’). Research investigating interpersonal incidental synchrony has found that individuals feel more bonded to others after performing a task with them that involves synchronous, as compared to asynchronous interpersonal movement. For instance, adults exhibit increased feelings of bonding, trust, empathy, and pro-social behaviours following interpersonal synchrony^12–16^. Adults are also more likely to imitate a partner after engaging in a synchronous movement task with them as compared to after performing those movements asynchronously with their partner^17^. Similarly, in children, interpersonal synchrony has been shown to facilitate pro-social sharing^18,19^ and helping behaviour^5^. Therefore, moving in coordination with other people seems to incur social interaction benefits. Synchrony and social bonding outcomes can in turn be influenced by more complex social structures such as identity, intergroup dynamics and representations of self and other^20–22^. Since movement synchrony helps forge and signal shared goals/intentions, group alliance and similarity, it has been proposed to play a prominent role in social interactions. What, then, are the mechanisms that link movement alignment in the form of visuomotor synchrony to positive social bonding outcomes? Two prominent accounts have been put forth in the literature to explain the synchrony—social bonding link: a bottom–up sensorimotor coupling account and a top–down joint action account.

Sensorimotor coupling defines the process whereby an external rhythm is identified (e.g., through visual observation) and integrated into one’s own movements^23^. Previous research has shown that interpersonal synchrony is improved when information about an external rhythm is available through multiple modalities, for example visual and auditory^24^. Further, some stimuli may be easier to couple with than others. For instance, viewing a biologically similar stimulus (i.e., hand), compared to a non-social, mechanical stimulus, can facilitate synchronisation^25^, arguably due to the ease of creating motor representations of the other person’s actions^26–30^. In turn, perceptual representation of synchronised movement may result in a less effortful and more rewarding experience through minimising neural processing costs^31^, thereby creating a cycle encouraging interpersonal synchrony. Indeed, an fMRI study has shown the brain’s reward system to be activated following synchronous, as compared to asynchronous, interpersonal movement^32^. Moreover, previous work has investigated whether the exact degree of synchrony predicts social bonding, as this would suggest that sensorimotor coupling between individuals is a key driver of interpersonal synchrony and its subsequent social bonding outcomes. The evidence has been mixed. While one study found that the degree of synchrony between individuals predicted subsequent feelings of affiliation^13^, more recent work has found that degree of coordination did not predict cooperation between individuals^33^. Thus, more research is needed to understand under which conditions the degree of synchrony is related to positive social interactional outcomes. Given that adults and children show greater social bonding to a partner following incidental synchrony^5,13^, a social context that encourages sensorimotor coupling may facilitate synchronisation accuracy and feelings of social closeness.

Top–down joint action processes can also facilitate interpersonal synchrony, and indeed modulate how quickly sensorimotor coupling takes place. Joint action can be defined as the coordination or complementarity of two or more individuals’ actions “to bring about a change in the environment” within a social interaction^34^. According to this framework, the shared goal and mutual knowledge that the goal is shared, unites the interaction partners’ intentions, attention, and representation of the task in hand^35^. These shared representations enable easier prediction of the other’s behaviour and coordination of movements^34,36^. Note that all instructed, uninstructed and incidental types of synchrony can occur within joint action contexts, depending on what the objects of people’s shared intentions are.

Prior research has indeed found that joint action contexts facilitate synchronisation, even when individuals share a goal or intention that is not related to synchronisation. For example, individuals who shared a mutual goal to empty a box of 100 plastic balls coordinated movements more than those without a shared goal^37^. Moreover, research has shown that sharing visual attention during a joint task can be sufficient to promote feelings of social closeness with a partner^38^. Other work has found that individuals with a shared intentionality to move in time with each other reported feeling closer to each other than those with a shared intentionality to synchronise with an auditory beat, i.e., when emergence of interpersonal synchrony was incidental^39^. The authors conclude that a combination of synchrony and shared intentionality to synchronise gives rise to the greatest social bonding outcomes. Additionally, when participants shared the intention to synchronise with each other, and the salience of a social context (i.e., synchronisation with visible actors vs point-light displays) was enhanced, even less precise forms of synchronisation elicited similar levels of cooperation^33^. Furthermore, recent work has found that when adults intentionally moved in time with a partner, they were more likely to imitate their partner’s actions, compared to a partner they had not moved in time with^17^. This work also found that when individuals incidentally moved in time with each other (i.e., were not instructed to synchronise) they were no more likely to imitate each other than those who did not incidentally synchronise. This suggests that a shared intention to synchronise may be fundamental for promoting imitation. Thus, beyond precise sensorimotor coupling, the social bonding outcomes of synchrony seem to depend on whether synchronisation occurs within a salient social context in which joint action can arise. Based on this research, we might expect that instructing a participant to synchronise with a partner would facilitate synchrony and social bonding outcomes. In light of this, the current study aims to investigate the impact of instruction on synchronisation accuracy within social and non-social contexts.

The facilitatory effect of a joint action context on synchronisation has been shown even in the absence of any explicit instruction to synchronise with the other person. In adults, studies have robustly shown that people tend to spontaneously entrain to others’ movements when walking^40^, swinging their legs^41^, clapping^42^ and rocking in chairs^43^. One study with children found that as young as 2.5-year-olds spontaneously synchronise their drumming and do so better when performing drumming with another person as opposed to with a robot^25^. These findings suggest humans have a natural propensity to synchronise with others, with synchronisation improving when both sensorimotor coupling and joint action are facilitated simultaneously. Still, we do not know whether social bonding outcomes would follow from uninstructed synchronisation in children and adults.

Across two studies, we investigated how adults (*Study 1*) and children (*Study 2*) synchronise their movements and subsequently bond with their interaction partner. Synchronisation was assessed with a finger-tapping task, with participants being randomly assigned to either the instructed or uninstructed condition. Combining the bottom–up and top–down accounts of interpersonal synchrony, we created a social context that included joint action (i.e., participant tapping together with the partner) and better opportunities for sensorimotor coupling (i.e., participant viewing the partner’s hand). This social context was contrasted to a non-social context that included neither joint action (i.e., participant tapping on their own while the partner is doing another task) nor enhanced sensorimotor coupling (i.e., participant viewing a moving ball). The adult study aimed to establish the effects of instruction within a social context on synchrony and social bonding, before investigating if similar results would also be found in children.

We made four predictions: (1) during a finger-tapping activity, children and adults will synchronise with the stimulus better in the social than in the non-social context, (2) children and adults will feel more bonded to their partner after completing the finger tapping task in a social as compared to a non-social context, (3) synchronisation accuracy and social bonding will be greater in the instructed compared to uninstructed condition, and (4) the degree of interpersonal synchrony in the social context will positively predict social bonding. The latter hypothesis would reveal the relative contributions of the top–down versus bottom–up processes—beyond performing a task in a joint action context, the precise degree of sensorimotor coupling would be associated with the social bonding effects observed after interpersonal synchrony. Given that children as young as 2.5 years of age are able to synchronise with external stimuli and show improvement in synchronisation within a social context^25^, we would not anticipate a different pattern of results for children compared to adults, however the children may be more variable^44^.

## Study 1: adults

### Methods

#### Participants

Forty-three adults (37 women, 6 men, M_age_ = 20.44), predominantly White, undergraduate students, participated in the current study. Students volunteered to participate in return for course credit. Data from three participants were removed due to experimenter error. Therefore, the final sample consisted of forty adults (34 women, 6 men, M_age_ = 20.38) evenly split into the instructed and uninstructed conditions. Post-hoc power analysis and the reported effect sizes (see “Results” section below) show that despite some variability, our sample size allowed for sufficient power to detect the main effects found in both studies, when the type I error rate set was to 0.05 (1 − β ≥ 0.9). Ethical approval for both *Study 1* and *Study 2* were granted by the School of Psychology ethics committee at the University of Nottingham, reference number: F1075R. The experiment was conducted in accordance with the ethical standards of the Declaration of Helsinki and GDPR. Informed consent was obtained from all participations prior to participation.

#### Design

A 2 (context: social vs non-social) × 2 (instruction type: instructed vs uninstructed) × 2 (stimulus tempo: 750 ms vs 1000 ms tempo) mixed design was used. All participants were presented with two social and two non-social trials, with the visual stimulus being presented at 750 ms and 1000 ms tempo within each condition. The two speeds (750 ms and 1000 ms) were used in order to maintain engagement with this repetitive task, and to prevent tempo practice effects, particularly as the same task was used with both children and adults. No specific hypotheses were made about the effects of the speed conditions, though they were nevertheless included in the analysis to control for any potential confounding effects speed might have on synchronisation ability. The between-group factor was type of instruction, in which half of the participants were explicitly instructed to synchronise with the visual stimulus whilst the remaining half were told to tap as they wish, without any emphasis on synchronising with the stimulus. Participants were randomly assigned to the instructed or the uninstructed condition. Order of the social and non-social trials as well as the stimulus tempi was fully counterbalanced. The dependent variables were synchronisation accuracy (i.e., difference from the stimulus in ms) and social bonding as measured by subjective assessment of proximity with the partner.

#### Materials and measures

##### Synchronisation accuracy

**Figure 1:**
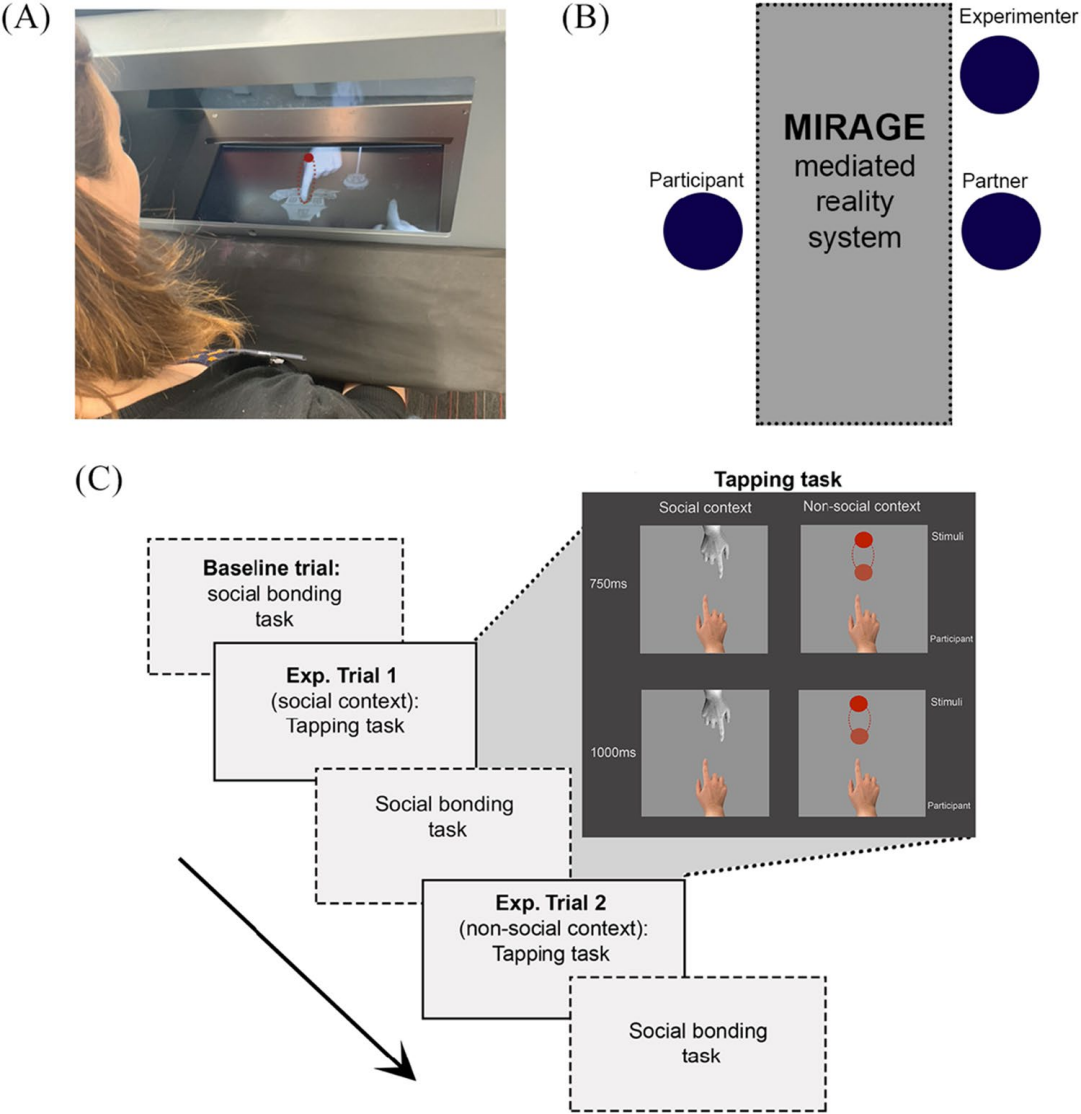
Schematic of the study procedure. (**A**) Participant view of the MIRAGE window, with their hand on the bottom right of the window and the social stimulus (partner’s hand) and non-social stimulus (red ball) opposite. The cartoon house and animal were used in the child-friendly dummy task. (**B**) Study set-up. The partner sat in the same position in both the social and the non-social context conditions. (**C**) Procedure of Study 1 and Study 2 (order of context and stimulus tempo counterbalanced across participants). In Study 1, participants completed two social context trials at each tempi and two non-social context trials at each tempi, therefore a total of four tapping trials. In Study 2, participants only completed one social trial and one non-social trial, with the tempo varied between trials (e.g. social at 750 ms and non-social at 1000 ms) for a total of two tapping trials.

The tapping tasks were conducted using a mediated reality device called MIRAGE (see Fig. 1A)^45,46^, which presents live video images of the participant’s hand in real time. When participants put their hand into the MIRAGE device, they can no longer see their actual hand; instead, they see a live footage (minimal delay of 16 ms) of their hand in the same spatial location, depth plane and from the same visual perspective as their ‘real’ hand. Each participant had their arm covered with a black curtain to prevent them from viewing their ‘real’ hand within the MIRAGE device. This device has been successfully used with adults and children in previous research^46–50^.

In our study, the social (hand) and non-social (moving circle) stimuli were presented in the MIRAGE window opposite the live image of the participant’s hand, as can be seen in Fig. 1A. Finger taps were registered using a button placed under the participant’s index finger. The MIRAGE device was connected to a computer that controlled the tempo of the visual stimulus and recorded the timestamps of the participants’ taps at millisecond precision. Each participant completed four trials in total: two trials in the social context (at 750 ms and 1000 ms tempo) and two trials in the non-social context (at 750 ms and 1000 ms tempo). Each trial consisted of 70 cycles of visual stimulus. Therefore, for each participant, approximately 140 responses were recorded for the social context and 140 for the non-social context.

Synchronisation error was calculated as the Root Mean Square (RMS) of the difference between the participant’s and the stimulus’ inter-tap intervals. The stimulus inter-tap intervals were constant over time within a trial (i.e., either 750 or 1000, depending on the stimulus tempo). To calculate the participants’ inter-tap intervals, we subtracted the timestamp of one tap from the timestamp of the subsequent tap in a sliding window fashion (window size = 2). Next, the difference between the participants’ inter-tap intervals and that of the stimulus was taken. Finally, the RMS of this difference was calculated, yielding one synchronisation error score per participant per trial. To obtain single synchronisation accuracy scores for the social and non-social conditions each, we took the mean of the two trials comprising these conditions, respectively. Synchronisation error scores closer to zero indicate less difference between the participant and the stimulus, and thus better synchronisation accuracy.

##### Social closeness

Social closeness was measured using the 7-point Inclusion of Other in Self scale^51^. In this scale, two circles labelled “you” and “[partner’s name]” were shown in differing proximities to each other across the 7 points, starting with very distant at 1 and gradually getting closer, with a near-merging image at 7. To calculate the change in feelings of closeness, a baseline measure was taken once at the start of the study and four more times at the end of each tapping trial. Change in social closeness was calculated separately for each experimental trial by deducting the participants’ baseline score from their score following that trial. Then, the mean of the two difference scores obtained from the social and the non-social trials were taken to construct one social and one non-social score per participant, respectively. The more positive the social bonding difference values are, the closer the participant felt towards the experimenter after the tapping task as compared to baseline.

#### Procedure

All participants were tested in a quiet room at The University of Nottingham, where they were sat across from the experimenter and the partner in both social and non-social context conditions (see Fig. 1B for a schematic of the set-up). The experimental procedure took a total of 30 min.

Firstly, participants completed a baseline measure of the Inclusion of Other in Self scale. Then, participants placed their dominant hand into the MIRAGE to start the finger tapping task. The participants were instructed to place the tip of their index finger on a button that was used to register taps. The button made a dull click when tapped. The participants were instructed to keep their hand in this location throughout all trials. All participants completed a practice trial to ensure they were comfortable with MIRAGE and that they understood the task. Once five consecutive taps had been successfully registered, the practice trial was terminated, and the participant proceeded to the experimental trials.

In the experimental trials, participants completed two social and two non-social context trials, each followed by the social closeness measure (see Fig. 1C). Regardless of the instruction condition, all participants were first given the following instructions: “If you look through the MIRAGE window, you will see a ball (non-social context) moving elliptically up and down or [the partner’s name]’s finger (social context) tapping. Please start tapping as soon as you see the ball/finger start and keep tapping until it stops”. At this point, the instructions diverged depending on the instruction condition. While participants in the instructed condition were told “to try to tap in time with the ball/finger as best as you can”, participants in the uninstructed condition were told “to keep looking at the ball/finger while tapping as slowly or as fast as you wish”.

In the non-social context, a red ball moved up and down in an elliptical movement for the duration of the trial. In the social context, participants viewed a pre-recorded video of a hand tapping although they were told that the partner was tapping in real time. The size of the area in which the visual stimulus moved was kept constant across the social and non-social stimuli to ensure that the amount of visual information was similar between conditions. In both social and non-social context, the partner sat opposite the participant, without explicitly interacting with the participant (e.g., by making eye contact or smiling). The only differences between contexts were that in the social context: (a) the participants were told that the partner was also finger-tapping, and (b) the participants viewed a hand, rather than a ball, as they tapped.

To ensure that all participants attended to the stimuli similarly in all conditions, we also introduced a dummy task. A child friendly dummy task was used so it could also be introduced in *Study 2*. Participants were told the visual stimulus was knocking on the door of the house (see Fig. 1A) and waking up the animals inside. Pictures of animals would then appear next to the visual stimulus 2–3 times per trial. Since the animal pictures appeared next to the target stimulus, participants were required to look towards the stimulus at all times and then tell the names of the animals whose pictures popped up.

### Results

#### Analysis

For both *Study 1* and *Study 2*, the timestamp data was processed using a custom-written script on MATLAB R2018b and the statistical analyses (including the power analysis using package “pwr”)^52^ were conducted using R^53^. To examine Hypotheses 1–3, mixed 2 (context: social vs non-social—within variable) × 2 (instruction: instructed vs uninstructed—between groups variable) × 2 (stimulus tempo: 750 ms vs 1000 ms—within variable) separate ANOVA tests were conducted with synchronisation accuracy and social bonding measures as the dependent variables. Stimulus tempo was included in the analyses as an independent variable despite not being part of a specific hypothesis to control for its potential effects on our outcome measures. To examine Hypothesis 4, linear regression was conducted within the social context, with synchronisation accuracy as the predictor and social bonding measure(s) as the outcome variable(s).

#### Synchronisation accuracy

##### Hypotheses 1 and 3

Adults will synchronise with the stimulus better in the social than in the non-social context and when instructed compared to uninstructed.

**Figure 2:**
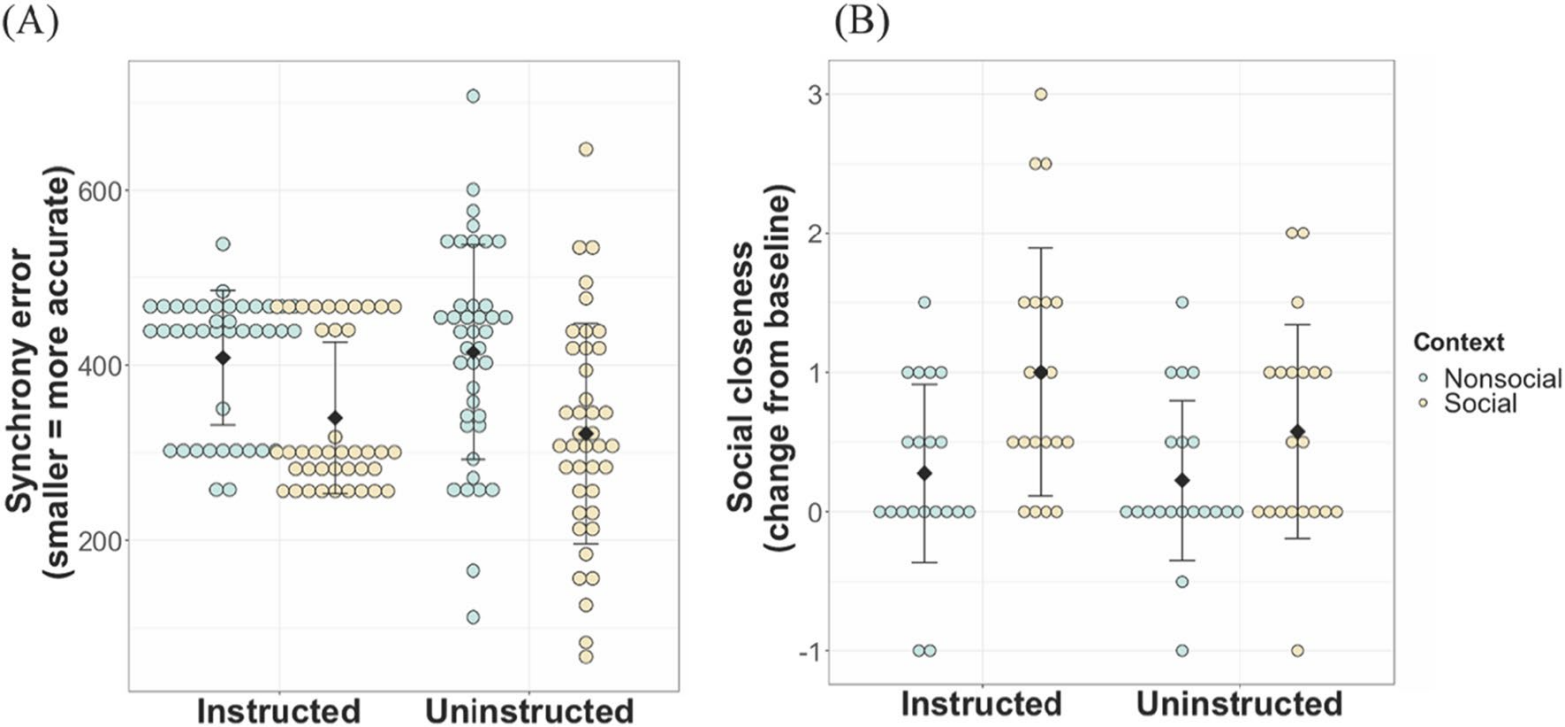
Synchronisation accuracy and social closeness by context and instruction conditions in adults. (**A**) Synchronisation accuracy per context and instruction condition. The y-axis shows mean RMS of the difference in inter-tap intervals between the participant and the stimulus, with higher scores indicating more errors and worse synchrony accuracy. (**B**) Social closeness per context and instruction condition. Mean change in closeness score (test-baseline), with higher scores indicating an increase in closeness as compared to baseline. Coloured dots indicate individual data points, black diamonds indicate group mean and whiskers indicate standard error of the mean.

A mixed 2 × 2 × 2 analysis of variance (ANOVA) was used to investigate the effects of context (social vs non-social), instruction (instructed vs uninstructed) and stimulus tempo (750 ms vs 1000 ms) on synchronisation accuracy (i.e., RMS of inter-tap intervals). The results revealed main effects of context (*F*(1,112) = 31.71, *p* < .0001, partia*l η*^*2*^ = 0.46), and tempo (*F*(1,112) = 85.83, *p* < .0001, partia*l η*^*2*^ = 0.70) on synchronisation accuracy. There was no significant main effect of instruction or any significant interactions (all *p*s > .05; see. Table S1 for details). Participants synchronised better (i.e., lower error) in the social context (*M* = 343.66, *SD* = 103.04) than in the non-social context (*M* = 408.67, *SD* = 97.38; see Fig. 2A) and better when the visual stimuli moved at 750 ms tempo (*M* = 319.81, *SD* = 93.52) as compared to at 1000 ms tempo (*M* = 431.70, *SD* = 84.68). These findings show that irrespective of whether explicitly instructed to do so, participants were better able to synchronise with the partner’s hand in the social context compared to the moving ball stimulus in the non-social context. To account for the possibility of more errors in cases when inter-tap intervals are larger (i.e., stimulus tempo at 1000 ms vs 750 ms), we also calculated a normalised synchronisation accuracy metric by dividing the RMS inter-tap intervals by the stimulus tempo. Analyses using this normalised metric similarly showed significantly better synchrony performance in the social than in the non-social context (see Table S2).

#### Social closeness

##### Hypotheses 2 and 3

Adults will feel more bonded to their partner after completing the finger tapping task in a social as compared to a non-social context and when instructed compared to when uninstructed.

To assess the impact of context (social vs non-social), stimulus tempo (750 ms vs 1000 ms), and instruction condition (instructed vs uninstructed) on change in social closeness from baseline, a mixed 2 × 2 × 2 ANOVA was employed. This analysis revealed a significant main effect of context (*F*(1,114) = 58.11, *p* < .0001, partia*l η*^*2*^ = 0.34), on social closeness. No other main effects or interactions were statistically significant (*p* > .05). While participants’ social closeness towards the partner increased from baseline levels (*M* = 5.90, *SD* = 0.98) in both the social (*M* = 5.08, *SD* = 1.20) and non-social (*M* = 5.68, *SD* = 1.05) contexts, as can be seen in Fig. 2B the increase was significantly higher in the social context (*M* = 0.82, *SD* = 0.87) as compared to the non-social context (*M* = 0.23, *SD* = 0.66). This suggests that social context, but not explicit instructions to synchronise, affected the participants’ feelings of social closeness.

##### Hypothesis 4

The degree of interpersonal synchrony in the social context will positively predict social bonding.

Finally, using linear regression analysis, we explored whether synchrony accuracy is associated with change in social closeness within the social context condition. The results were not significant (*F*(1,38) = 0.92, *β* = 0.003, *SE* = 0.003, *p* = .34, *R*^2^ = 0.001), indicating that the degree of synchronisation with the partner was not related to the observed increase in feelings of social closeness.

### Discussion

As predicted, we found that a social context facilitated synchronisation accuracy and social bonding with an interaction partner in adults. This novel finding in adults corresponds to previous work that has demonstrated social facilitation of synchronisation accuracy in children^25^. Contrary to our predictions, synchronisation accuracy was not enhanced when the participants were explicitly instructed to tap in time with the stimulus, and the degree of interpersonal synchrony within the social context did not predict subsequent social bonding.

Despite the partner being present in both the social and non-social contexts, the social context enhanced synchronisation accuracy and social bonding significantly more than non-social context did. This finding suggests that both synchronisation accuracy and social bonding can be facilitated (a) in a context in which the partner is perceived to be engaging in the task, and (b) when the participant views a biologically more similar stimulus that facilitates sensorimotor coupling. Given that our social context combined these two elements, we cannot identify specifically which aspect of the social context led to the observed effects. Nevertheless, these findings extend previous work that has found increased social bonding following instructed interpersonal synchrony^13,14,54^ by showing that the same effects are observed following uninstructed synchrony as well.

The hypothesised effect of instruction on synchronisation accuracy was not observed; participants were similarly accurate at synchronising with the visual stimulus in the instructed and uninstructed conditions. Yet, visual inspection of the data shows that participant responses were less variable in the instructed condition than in the uninstructed condition, with the responses in the instructed condition displaying a bipolar distribution, suggesting a clustering of the errors based on the stimulus tempo. The absence of an effect of instruction seems to contradict previous work demonstrating that intentionality facilitates synchronisation^55,56^. However, in the current study, the intention was not explicitly shared between the participant and the partner, whilst in previous work the intention was shared between individuals^55^. This may suggest that shared intentionality between individuals, rather than individual intention, is key to facilitating coordination, rather than merely the instruction to synchronise.

Additionally, we found no relationship between the degree of synchronisation accuracy and the strength of feelings of social closeness. This may suggest that sensorimotor coupling of visual information did not drive the bonding effects; instead, joint action factors within the social context may have been sufficient to boost social closeness. These contradictory results are likely to be explained due to the differences in the social bonding outcome measures that were utilised. Feelings of affiliation or social closeness may therefore be driven by sensorimotor coupling, whilst cooperation may not. Alternatively, it is possible that the low sensitivity of the social closeness scale to detect change in social bonding could explain why no relationship with synchrony was found^57^. For instance, the Inclusion of Other in Self (IOS) Scale used may not capture affective, expressive or implicit aspects of social bonding. Instead, it is argued the IoS measures explicit perceptions of shared similarity and closeness^51,57^. Within the current study, this may suggest that sensorimotor coupling does not drive explicit reports of affiliation, but future work is needed to understand if the same is true for other aspects of prosocial behaviour.

Finally, despite not having any specific hypothesis based on stimulus tempo, we also found that visuomotor synchronisation accuracy was higher with stimuli moving at a faster tempo. This effect is most likely attributable to the Spontaneous Motor Tempo of individuals being faster than the tempo at which the stimuli was presented. Previously work has found that adults aged 18–38 years-old have a mean spontaneous motor tempo of 630 ms^58^. It is therefore likely that participants were more comfortable tapping at the faster rate of 750 ms than they were at 1000 ms, therefore synchronisation accuracy was greater when the tempo of the stimuli was faster. Moreover, previous work has found that uninstructed synchrony with non-social visual stimuli becomes more likely to occur as the difference between individual preferred tempo and stimulus tempo decreases^59^. With regards to the current study, this may provide further explanation as to why tempo significantly impacted both uninstructed and instructed synchrony.

Social skills and sensorimotor ability develop significantly throughout childhood and adolescence^44,47,60–62^. As demonstrated in previous work and in the results of *Study 1*, interpersonal synchrony plays a significant role in social interactions. We conducted *Study 2* to explore whether children show similar facilitation of synchrony and social bonding within a social context. We also sought to investigate whether synchrony performance predicts social bonding effects.

## Study 2: children

### Methods

#### Participants

Sixty-one children (30 girls, 31 boys) aged 4.53 to 14.32 years old (*M*_*age*_ = 8.79, *SD* = 2.18) from predominantly White, middle class families took part in the present study. The participants were recruited through a public event held at The University of Nottingham in July 2019, in which children participate in short research studies over the course of a week. As we were running the study at a public event, we aimed to recruit a minimum of 20 participants per condition, and as many as possible thereafter. Three participants with a confirmed clinical diagnosis of autism spectrum disorder were removed from analysis as previous work has found this population to exhibit atypical synchrony behaviour^63–65^. Four participants were removed from analysis for being extreme outliers (± 2 SDs from the mean). Therefore, the final sample consisted of 54 neurotypical children (27 girls, 26 boys) aged 4.53 to 14.32 years old (*M*_*age*_ = 8.80, *SD* = 2.16). There were 26 participants in the instructed condition and 28 participants in the uninstructed condition. All children were screened for developmental difficulties via a parental background questionnaire. Verbal mental age was assessed using the British Picture Vocabulary Scale III (BPVS III)^66^ to ensure participants did not have a delay in their cognitive development. The guardians of all children gave informed written consent for their child to participate and the children provided verbal assent at time of the study.

#### Design

The design of *Study 2* differed slightly from *Study 1* due to adjustments made for a child sample. Participants completed two, rather than four, tapping trials in total: one in the social context and one in the non-social context. This meant that stimulus tempo and the order of context conditions were counterbalanced across, not within, participants, such that half of the participants were presented with the social stimulus at 750 ms and the non-social at 1000 ms, while the remaining half were presented with the social stimulus at 1000 ms and the non-social at 750 ms. The independent variables of this study were identical to *Study 2*: context (social vs non-social), instruction type (instructed vs uninstructed) and stimulus tempo (750 ms vs 1000 ms). The dependent variables were synchronisation accuracy (i.e., difference from the stimulus in ms) and social bonding. Social bonding was measured in two ways: change in social closeness, measured by subjective assessment of proximity with the partner and change in spontaneous behavioural mimicry, measured by frequency of mimicked behaviours. The mimicry task was added as a child-friendly way of assessing social bonding. The nature of the testing event in which *Study 2* was conducted allowed for the addition of the spontaneous behavioural mimicry.

#### Materials and measures

##### Synchronisation accuracy

To measure synchronisation accuracy, the same MIRAGE virtual reality device and the same measure of RMS of inter-tap intervals was used as in *Study 1*.

##### Social closeness

A child-friendly version of the social closeness task was used here as compared to *Study 1* to ensure participants could understand and relate to the task. To assess social closeness, the participants were asked to imagine a hypothetical scenario (e.g., waiting for a train at the station) and indicate first where they would like to sit, and then where they would like the partner to sit in this scenario by placing stickers that represented the child and the partner. The scenarios were accompanied by matching pictures that had 7 seats on them. Building upon similar measures that used physical closeness as a proxy for social closeness^22,67,68^, participants placing themselves closer to the partner was considered indicative of higher social closeness. Since the children were first asked to place themselves and then the partner, the distance between the two people was calculated as a proportion of the number of available seats once the children had placed themselves. To examine change in social closeness, the baseline distance score was subtracted from the distance scores following the tapping trials. Therefore, positive values indicated increased closeness from baseline, while negative values indicated decreased closeness.

##### Mimicry

Based on research showing positive affiliative effects of mimicking and being mimicked^69,70^, spontaneous behavioural mimicry was assessed as an indicator of social bonding. Mimicry was assessed three times with a two-minute-long, semi-structured picture-guessing game played between the participant and the partner: first at baseline, and twice more thereafter each tapping trial. In the game, the partner viewed three pictures in sequence and described each to the participant. The participant, who had not seen the pictures, then guessed which picture (out of three options per picture) they thought the partner had been describing. Whilst describing the pictures, the partner made upper body touches on three areas (i.e., rubbing or scratching her head, shoulder or upper arm), amounting to a total of nine touches per block. The same picture descriptions were used across participants with the touches occurring at the same timepoints.

The frequency of spontaneous behavioural mimicry was post-hoc coded from the video recordings of the mimicry sessions using the open-source E-LAN software (version 5.8)^71^. An action was considered an instance of mimicry if the children touched, rubbed or scratched their head, shoulders or upper arms following the partner performing one of these actions within the same block. Two raters, one blind to the hypotheses and both blind to the actions of the partner, coded the videos. The primary rater coded 100% of the videos and the secondary rater, who was blind to the hypotheses of the study, coded 40% of the videos. A Pearson's correlation test on the frequency values per participant revealed excellent inter-rater reliability (*r*(*25*) = 0.89, *p* < .0001), and the primary rater's codes were used for the few cases where there was disagreement between the raters. To examine change in mimicry, difference scores were obtained by deducting the frequency of mimicry at baseline from the frequency of mimicry following each tapping trial.

#### Procedure

The procedure was identical to *Study 1* with the addition of the spontaneous behavioural mimicry measure completed at baseline and following each tapping trial.

### Results

#### Synchronisation accuracy

##### Hypotheses 1 and 3

Children will synchronise with the stimulus better in the social than in the non-social context and when instructed compared to uninstructed.

**Figure 3:**
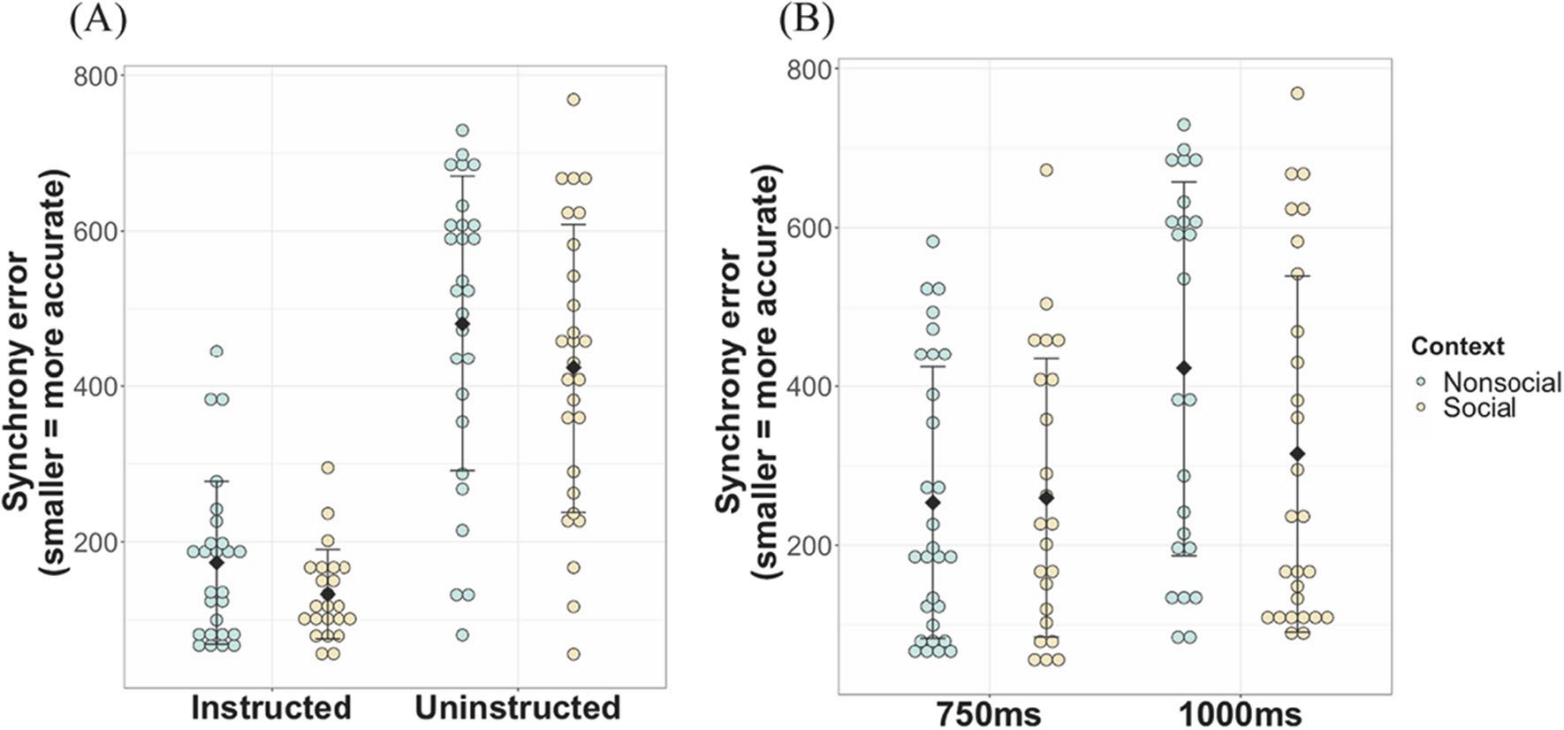
Synchrony accuracy by context, stimulus tempo and instruction conditions in children. Lower scores on the y axis indicate less synchrony errors and better accuracy. Coloured dots indicate individual data points, black diamonds indicate group mean and whiskers indicate standard error of the mean. (**A**) Synchronisation accuracy per context and instruction condition. (**B**) Synchronisation accuracy per context and stimulus tempo conditions.

A mixed 2 × 2 × 2 ANOVA was used to investigate the effects of context (social vs non-social—within variable), instruction (instructed vs uninstructed—between groups variable) and stimulus tempo (750 ms vs 1000 ms—within variable) on synchronisation accuracy (i.e., RMS of inter-tap intervals). The results revealed significant main effects of context (*F*(1,45) = 9.00, *p* = .004, partia*l η*^*2*^ = 0.17), instruction (*F*(1,47) = 84.15, *p* < .0001, partia*l η*^*2*^ = 0.64) and tempo (*F*(1,45) = 41.98, *p* < .0001, partia*l η*^*2*^ = 0.48). As can be seen in Fig. 3A, participants’ synchronisation accuracy was higher (i.e., lower error) in the social context (*M* = 289.54, *SD* = 202.77) than in the non-social context (*M* = 330.02, *SD* = 217.60). Participants also synchronised better in the instructed condition (*M* = 154.22, *SD* = 87.27) than in the uninstructed condition (*M* = 452.06, *SD* = 188.07) and when the stimulus moved at 750 ms (*M* = 256.25, *SD* = 170.72) than at 1000 ms (*M* = 365.55, *SD* = 233.45), see Fig. 3B. There was a significant interaction between instruction condition and tempo *F*(1,45) = 16.56, *p* = .0002, partia*l η*^*2*^ = 0.27), such that within the uninstructed condition, synchronisation accuracy was better when the stimulus tempo was 750 ms (*M* = 268.59, *SD* = 188.41) than when it was 1000 ms (*M* = 443.99, *SD* = 254.76; *F*(1,25) = 31.75, *p* < .0001), while no such difference was observed within the instructed condition (*F*(1,22) = 1.94, *p* = .18). No other interaction effect was significant (all *p*s > .05; see. Table S3 for details).

Similar to the approach followed in *Study 1*, we also assessed the normalised synchronisation accuracy metric, which was calculated by dividing the RMS of inter-tap intervals by the stimulus tempo for that trial. These results confirmed that even when accounting for the possibility of increased errors in larger inter-tap intervals, the effect of context remained significant such that children synchronised with the stimulus better in the social than in the non-social context (see Table S4).

#### Social closeness

##### Hypotheses 2 and 3

Children will feel more bonded to their partner after completing the finger tapping task in a social as compared to a non-social context and when instructed compared to when uninstructed.

**Figure 4:**
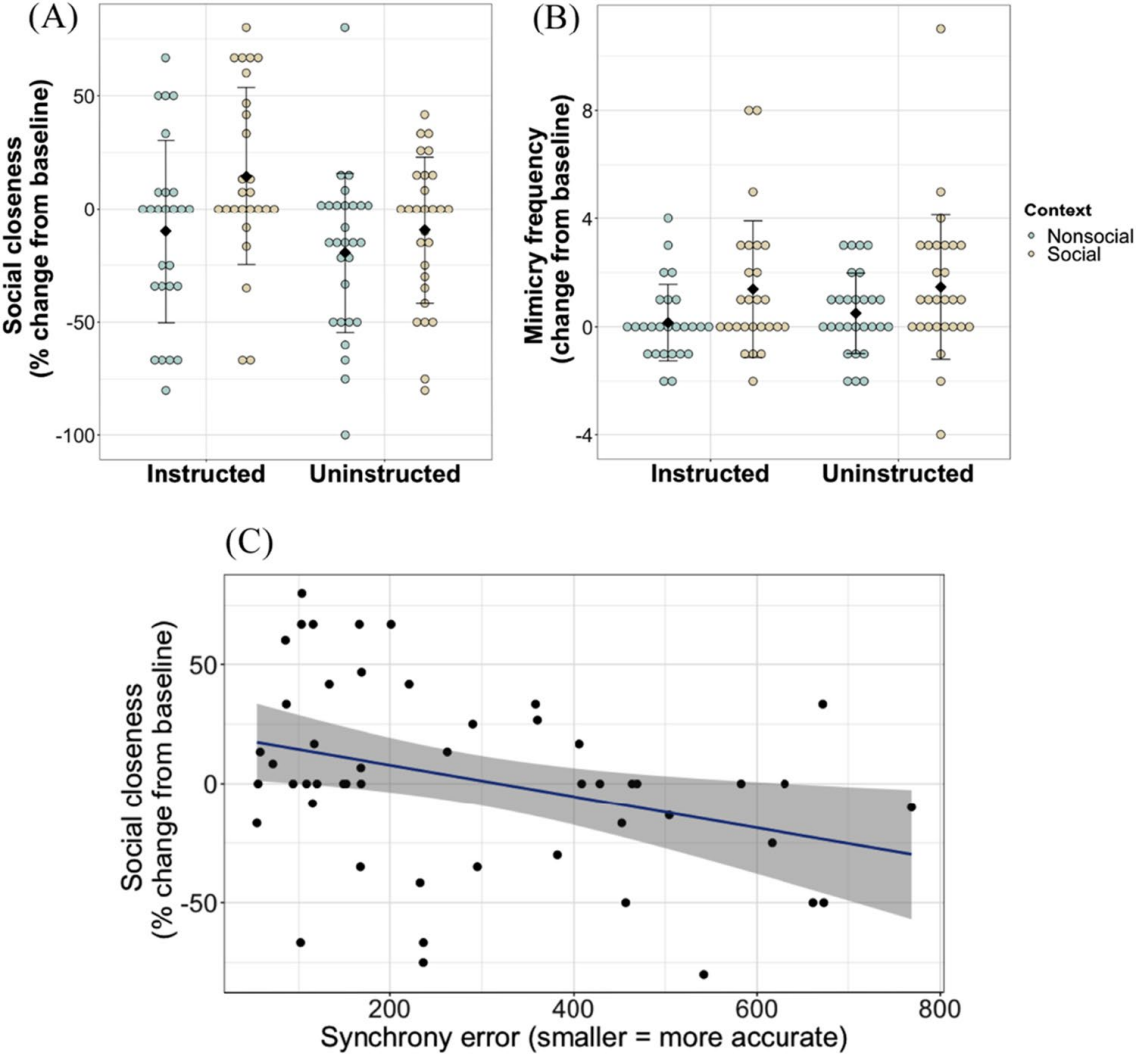
Social bonding by context, instruction and synchronisation accuracy in children. In (**A**) and (**B**), coloured dots indicate individual data points, black diamonds indicate group mean and whiskers indicate standard error of the mean. (**A**) Change in social closeness (test-baseline) per context and instruction condition, with positive scores indicating an increase and negative scores indicating a decrease in closeness as compared to baseline. (**B**) Change in mimicry frequency (test-baseline) per social context and instruction condition, with positive scores indicating an increase and negative scores indicating a decrease in mimicry as compared to baseline. (**C**) Relationship between synchrony error and change in social closeness within the social context. Shading indicates 95 confidence intervals.

At baseline, the children preferred, on average, to put 52.02% distance (*SD* = 27.78) between themselves and their partner. This distance decreased to 50.74% on average (*SD* = 30.74) in the social context and increased to 60.93% on average (*SD* = 30.26) in the non-social context. A mixed 2 × 2 × 2 ANOVA was used to examine the effects of context (social vs non-social), instruction (instructed vs uninstructed) and stimulus tempo (750 ms vs 1000 ms) on change in social closeness (i.e., difference from baseline). This analysis revealed a significant main effect of context on social closeness (*F*(1,50) = 11.46, *p* = .002, partia*l η*^*2*^ = 0.19). There was no significant main effect of instruction or stimulus tempo and no interaction effects (all *p’s* > 0.05). As can be seen in Fig. 4A, while social closeness towards the partner increased following tapping in the social context (*M* = 2.13, *SD* = 37.41), a decrease was observed in feelings of closeness following tapping in the non-social context (*M* = -14.85, *SD* = 37.69).

#### Mimicry

On average, the children mimicked their partner’s mannerisms 0.89 times (*SD* = 1.08) at baseline, 2.45 times (*SD* = 2.58) after performing the finger-tapping task in the social context and 1.19 times (*SD* = 1.50) after performing the finger-tapping task the non-social context. The percentage of children who mimicked their partner at least once was, 52% (n = 28) at baseline, 78% (n = 42) in the social context condition, and 61% (n = 33) in the non-social context condition.

A mixed 2 × 2 × 2 ANOVA was used to investigate the effects of context (social vs non-social), instruction (instructed vs uninstructed) and stimulus tempo (750 ms vs 1000 ms) on change in mimicry frequency (i.e., difference from baseline). The results revealed a main effect of context on mimicry frequency (*F*(1,50) = 14.90, *p* = .0003, partia*l η*^*2*^ = 0.23). There was no significant main effect of instruction, stimulus tempo or any interaction effects (all *p’s* > 0.05). As can be seen in Fig. 4B, while mimicry frequency showed an increase from baseline in both social and non-social context conditions, this increase was significantly greater in the social context (*M* = 1.72, *SD* = 2.69) as compared to the non-social context (*M* = 0.34, *SD* = 1.47).

##### Hypothesis 4

The degree of interpersonal synchrony in the social context will positively predict social bonding.

Two separate linear regression tests were used to investigate whether synchronisation accuracy within the social context condition predicted change in spontaneous behavioural mimicry and social closeness. While synchronisation accuracy did not predict change in mimicry frequency (*F*(1,45) = 0.29, *β* = 0.001, *SE* = 0.002, *p* = .59, *R*^2^ = 0.005), it significantly predicted change in social closeness (*F*(1,48) = 20.47, *β* = 0.08, *SE* = 0.02, *p* < .0001, *R*^2^ = 0.28), see Fig. 4C.

### Discussion

As predicted, and in line with previous works^6,25^, we found that both visuomotor synchrony and social bonding were facilitated in a social as compared to a non-social context. Extending previously used measures of social bonding, we showed that children spontaneously mimicked their partner more following interpersonal synchrony compared to synchronisation in the non-social context.

In line with Hypothesis 3, children synchronised with significantly greater accuracy when explicitly instructed to synchronise with the stimulus compared to when they were left free to tap as they wished. Unlike the adults in *Study 1*, children spent longer participating in the study, participated in more tasks and had more opportunities to socially engage with the experimenter. This may have had a heightened sense of commitment when instructed, and hence increased effort to synchronise^72^. In addition, prior work has demonstrated children are less consistent in how they tap as compared to adults^73,74^, so it is possible that an explicit instruction therefore increased effort and improved synchronisation accuracy in the instructed condition.

Additionally, despite lower synchronisation in the uninstructed condition, social bonding for both measures remained similar across uninstructed and instructed conditions. This may suggest that merely completing the task together with the partner allowed for social bonding to occur. Yet, in line with Hypothesis 4, the degree of interpersonal synchrony still mattered: the more synchronous the children were with the partner, the greater was their feelings of social closeness. In comparison to adults, this may suggest that children are more likely to base social bonding on unconscious precise sensorimotor coupling between themselves and a partner.

Similar to the adult data, we found an unexpected effect of stimulus tempo on synchronisation accuracy. It is likely that a faster spontaneous motor tempo of children led to greater synchronisation accuracy in the 750 ms tempo compared to the 1000 ms tempo^58,59^.

### General discussion

Across two studies, we found support for our hypotheses that a social context, as compared to a non-social context, facilitated interpersonal synchrony (Hypothesis 1) and social bonding (Hypothesis 2) in both adults (*Study 1*) and children (*Study 2*). Contrary to our hypothesis (Hypothesis 3), being instructed did not facilitate synchronisation accuracy in the adult sample, but instruction did facilitate synchrony in children. Instruction did not facilitate social bonding in either sample. Uniquely in children, we found that the magnitude of social closeness was positively linked to the degree of synchronisation accuracy (Hypothesis 4).

We found evidence of social context facilitating interpersonal synchrony in adults and children. Previous paradigms have been criticised for their use of social and non-social contexts as often the social condition holds more sensory information, therefore allowing for more accurate synchronisation^25^. For instance, the available sensory information is much richer viewing the movement of a whole body as compared to a flashing dot or metronome. However, in the current study only the image of a hand was used as the social context, therefore the area covered by the moving visual stimuli was the same between conditions. Subsequently, this social facilitation effect is unlikely to be attributable to the amount of visual information. Instead, viewing a finger tapping may have enhanced neural representation of the external stimulus (i.e., hand movement), therefore increasing predictability and coordination with the stimulus^26–28^, making it easier to synchronise with. According to predictive coding principles, synchronisation with the social stimulus may have been less cognitively demanding and effortful, therefore producing fewer errors^31^. Furthermore, this current finding corresponds to previous works that have suggested the presence of a social agent can induce perceptions of a joint action^6,25^. Such joint action formation is argued to allow for better anticipation of movement of the external stimulus^34^ and therefore give rise to a coupling of self and other movement^20^.

Our social context differed from the non-social context both in terms of providing better opportunities for participants to couple with the visual stimulus (i.e., with a hand as opposed to a ball), and in terms of the partner engaging in joint action with the participants. Both of these factors may facilitate social bonding through promoting participants’ perception of the partner as *moving* like them and *being* like them^75^. We found that only in children, the degree of synchronisation accuracy was positively associated with feelings of social closeness. This indicates that precise sensorimotor coupling provided an additional cue for children's feelings of bonding, whereas for adults, this was irrelevant and/or insufficient. For instance, as the accuracy of synchronisation was not consequential to task success, for adults, it may have reduced the extent that accuracy impacted upon feelings of social closeness. Alternatively, children may have perceived being in time as more rewarding which may have enhanced synchrony and subsequent social bonding. However, as the children were much more variable in their synchronisation errors compared to the adults, it is harder to draw comparisons between the samples. Future research could seek to contrast joint-action and sensorimotor coupling contexts, for instance, by telling the participants that the non-social stimulus that they are synchronise with is a reflection of a partner’s movement, thus retaining a joint action context but removing sensorimotor coupling opportunities of a corporeal stimulus to disentangle the relative roles of bottom–up and top–down factors in facilitating social bonding following synchrony.

In addition, children mimicked the partner more following interpersonal synchronisation. Whilst this corresponds to previous research that has shown mimicry can facilitate social bonding^69,70^, the present finding corresponds with previous work that has found interpersonal coordination can increase imitation^17^. This expands possible measures of social bonding to include mimicry. Although interpersonal synchrony enhanced both social closeness and mimicry, the two aspects are distinguishable as synchronisation accuracy did not predict a change in mimicry. One reason for this may be the relatively reduced variability in the mimicry frequencies compared to the social closeness scores, yielding a correlational approach less powerful. Still, the absence of a link between mimicry and interpersonal synchrony suggests that while social closeness may be more selectively boosted by interpersonal synchrony, merely completing the tapping task with the partner appears to be sufficient for perceived coordination and increased mimicry to occur.

This study has emphasised the importance of interpersonal synchrony for social bonding, which opens up further questions as to whether an impaired ability to synchronise, especially in social contexts, may be related with social-communicative impairments. For instance, individuals with Autism Spectrum Conditions, Schizophrenia, social anxiety disorders and Attention Deficit and Hyperactivity Disorder have been found to exhibit differences in interpersonal synchrony^63–65,76–79^. Future work could therefore explore the relationship between sensorimotor processing and joint action mechanisms to further understand the observed differences in interpersonal synchrony in individuals with these disorders.

Whilst the present study provides key insights into the roles of instruction, context, and tempo on visuomotor synchronisation and social bonding, certain limitations should be noted. Firstly, in *Study 2* not all participants experienced both contexts at both tempi due to time constraints of the event at which the experiment was conducted. While it is unlikely that groups of children would be distinguished by their ability to synchronise better at social-fast vs social-slow stimuli, thereby affecting the results in a systematic way, future research can implement full counterbalancing to eliminate this possibility. Finally, the social vs non-social contrast in the current study was created by combining insights from the bottom–up sensorimotor accounts with the top–down joint action accounts. Future research can tease apart how either factor affects synchrony accuracy and its subsequent bonding effects.

The current research adds to a growing body of literature by revealing the importance of social context for interpersonal synchronisation and social bonding in children and adults. Interestingly, we show how social bonding can be elicited following spontaneous (uninstructed) interpersonal synchronisation. These findings provide an evidence-base that social contexts with increased opportunities for sensorimotor coupling and joint action improve synchronisation and bonding between partners.

## Supplementary Information

Supplementary Tables.
