# Supplementary material for: Social context facilitates visuomotor synchrony and bonding in children and adults
Source: Sci Rep. 2021 Nov 24;11:22869. doi: 10.1038/s41598-021-02372-2 (PMC8613228; doi:10.1038/s41598-021-02372-2)
Supplement: Supplementary file 1 — Supplementary Tables. [file 41598_2021_2372_MOESM1_ESM.docx]

**Supplementary Materials**

| *Table S1. Table outlining the non-significant results from Study 1.* | |
| --- | --- |
| **Test** | **Result** |
| ANOVA main effect of instruction on synchronisation accuracy | F(1,37)=0.15, *p*=0.7 |
| ANOVA interaction between context and tempo on synchronisation accuracy | F(1,112)=0.75, *p*=0.39 |
| ANOVA interaction between context and instruction on synchronisation accuracy | F(1,45)=0.009, *p*=0.92 |
| ANOVA interaction between tempo and instruction on synchronisation accuracy | F(1,112)=0.52, *p*=0.47 |
| ANOVA interaction between tempo, instruction and context on synchronisation accuracy | F(1,112)=1.08, *p*=0.30 |
| ANOVA main effect of instruction on social closeness | F(1,38)=1.79, *p*=0.19 |
| ANOVA main effect of tempo on social closeness | F(1,114)=0.006, *p*=0.94 |
| ANOVA interaction between context and tempo on social closeness | F(1,114)=0.52, *p*=0.47 |
| ANOVA interaction between tempo and instruction on social closeness | F(1,114)=0.16, *p*=0.69 |
| ANOVA interaction between tempo, instruction and context on social closeness | F(1,114)=0.006, *p*=0.93 |

| *Table S2. Results of a* mixed 2x2x2 ANOVA investigating the effects of context (social vs non-social) and instruction (instructed vs uninstructed) on the normalised synchronisation accuracy metric (i.e., RMS inter-tap intervals divided by the stimulus tempo) in adults (Study 1). | | | | |
| --- | --- | --- | --- | --- |
| **Predictor** | DF | **SS** | **F value** | **p value** |
| Context  (social vs non-social) | 38 | .38 | 38.26 | < .0001 |
| Instruction  (instructed vs uninstructed) | 59 | .002 | .10 | .76 |
| Context * Instruction | 59 | .01 | 1.16 | .28 |

| *Table S3. Table showing the non-significant results from Study 2.* | |
| --- | --- |
| **Test** | **Result** |
| ANOVA interaction between instruction and context on synchronisation accuracy | F(1,45)=0.11, *p*=0.74 |
| ANOVA interaction between context and tempo on synchronisation accuracy | F(1,47)=0.48, *p*=0.49 |
| ANOVA interaction between context, tempo and instruction on synchronisation accuracy | F(1,47)=0.19, *p*=0.66 |
| ANOVA main effect of instruction on social closeness | F(1,50)=3.68, *p*=0.06 |
| ANOVA main effect of tempo on social closeness | F(1,50)=1.08, *p*=0.30 |
| ANOVA interaction between context and tempo on social closeness | F(1,50)=0.81, *p*=0.37 |
| ANOVA interaction between context and instruction on social closeness | F(1,50)=2.58, *p*=0.11 |
| ANOVA interaction between context, instruction and tempo on social closeness | F(1,50)=0.028, *p*=0.87 |
| ANOVA main effect of tempo on mimicry | F(1,50)=0.12, *p*=0.87 |

| *Table S4. Results of a* mixed 2x2x2 ANOVA investigating the effects of context (social vs non-social) and instruction (instructed vs uninstructed) on the normalised synchronisation accuracy metric (i.e., RMS inter-tap intervals divided by the stimulus tempo) in children (Study 2). | | | | |
| --- | --- | --- | --- | --- |
| **Predictor** | DF | **SS** | **F value** | **p value** |
| Context  (social vs non-social) | 41 | .07 | 4.77 | .03 |
| Instruction  (instructed vs uninstructed) | 43 | 2.36 | 67.11 | < .0001 |
| Context * Instruction | 43 | .07 | 1.87 | .18 |
